# Internet-based UP-A intervention on treatment of stress, anxiety, depression, and psychological flexibility among adolescents with sub-clinical diagnosis of emotional disorders during the COVID-19 pandemic: a clinical trial

**DOI:** 10.1186/s40359-024-01735-4

**Published:** 2024-10-02

**Authors:** Vahideh Javadi, Farzaneh Ahmadi, Leila Salek Ebrahimi, Mohsen Dadashi, Seyedeh Elnaz Mousavi

**Affiliations:** 1 Department of Clinical Psychology, School of Medicine, Zanjan University of Medical Sciences, Zanjan, Iran; 2 Department of Biostatistics and Epidemiology, School of Medicine, Zanjan University of Medical Sciences, Zanjan, Iran; 3 Research Center of Psychiatry and Behavioral Sciences, Tabriz University of Medical Sciences, Tabriz, Iran; 4 Department of Clinical Psychology, Faculty of Medicine, Zanjan University of Medical Sciences, Zanjan, Iran

**Keywords:** Transdiagnostic therapy, anxiety, Depression, Psychological flexibility, Adolescents, Online therapy, COVID-19

## Abstract

This clinical trial aims to assess the effectiveness of internet-based Unified Protocol for Transdiagnostic Treatment of Emotional Disorders in Adolescents (UP-A) during the COVID-19 pandemic in reducing stress, anxiety, and depression, and psychological flexibility. 40 adolescents with subclinical features of emotional disorder randomly divided into two groups of intervention and control. The participants first completed DASS-21 and AAQ-2 questionnare online. Then, the intervention group received 12 sessions of UP-A through video calls on WhatsApp, 2 days per week each for 45 min. UP-A is an emotion-focused, cognitive-behavioral therapy consisting of 5 core modules or components that target temperamental characteristics, particularly neuroticism and resulting emotion dysregulation. Eventually the stress, anxiety, and depression levels decreased in intervention group and their psychological flexibility increased immediately and 3 months after the intervention.

**Clinical trial registration** This study was registered by Iranian Registry of Clinical Trials (Prospective, ID: IRCT20210428051113N1, Registration date: 14/06/2021; https://en.irct.ir/trial/55900).

**Supplementary Information:**

The online version contains supplementary material available at 10.1186/s40359-024-01735-4.

## Introduction

Adolescence is one of the most important stages of life when, along with physical changes, a series of changes occur in a person’s emotions, feelings, desires and imaginations. In this regard, emotional disorders such as anxiety, depression, and stress are common in adolescents. Emotional disorders are highly correlated with each other. Adolescents who experience anxiety symptoms are more likely to be at risk for an anxiety disorder or depression [37]. Co-occurrence of anxiety and depression has been reported in more than 50% of patients [1]. Unfortunately, most adolescents who suffer from an emotional disorder do not receive treatment of any kind. Further attention needs to be paid to symptoms of emotional disorders in adolescents and factors that play a mediating role in the formation or persistence of these disorders. Another issue that plays an important role in adolescents’ mental health is psychological flexibility. It is defined as being in contact with the present moment, fully aware of emotions, sensations, and thoughts, welcoming them, including the undesired ones, and moving in a pattern of behavior in the service of chosen values [30]. In other words, it means accepting our own thoughts and emotions and acting on long-term values rather than short-term impulses, thoughts, and feelings that are often linked to experiential avoidance and a way to control unwanted inner events [18]. Studies on healthy adolescents have shown that psychological inflexibility is correlated with greater symptoms of emotional disorders as well as lower self-esteem [8, 22, 38], lower quality of life, poorer social skills, and lower academic competence [17]. Because of the negative implications of psychological flexibility in adolescents, it is important to target psychological flexibility in treatments for adolescence.

Over the last few decades, some interventions like neurofeedback, transcranial direct current stimulation (tDCS), repetitive transcranial magnetic stimulation (rTMS) which target neural mechanisms and modulating brain activity and cortical excitability, have been effectively applied to depression and negative emotions in adolescents [3, 29], [42]. Also diagnosis-specific cognitive-behavioral approaches have been recognized as evidence-based approaches for treatment of emotional disorders. However, these approaches have limitations, such as insufficient attention to comorbidities, multiple underlying theories for each specific diagnosis, multiple treatment protocols, patients’ limited access to the most effective treatment, and the difficulty involved in specialist training [21, 28]. In this regard, a new generation of cognitive-behavioral approaches has been introduced based on the concept of transdiagnosis in the last two decades [6] Transdiagnostic approaches emphasize the commonalities between emotional disorders rather than their differences. They seek to treat comorbid disorders through the development of new and unified therapeutic protocols. In a systematic review and meta-analysis, Newby et al. [26] reported that transdiagnostic treatments are effective for reducing anxiety, and may be superior for reducing depression. One of the transdiagnostic approaches is the unified protocol for transdiagnostic treatment of emotional disorders (unified protocol: UP). It is an emotion-focused, therapy consisting of 5 core modules or components that target temperamental characteristics, particularly neuroticism and resulting emotion dysregulation, underlying all anxiety, depressive, and related disorders. These modules are preceded by a module focused on enhancing motivation as well as an introductory module on the adaptive nature of emotions that provides a framework for understanding emotional experiences. The UP contains cognitive reappraisal and exposure strategies, but the focus is on the reactions to the experience of emotion itself [7]. The results of studies support the efficacy of UP in the treatment of emotional disorders in Iran and other countries [2, 10, 14, 15, 23, 32]. The UP for Adolescents (UP-A) is a recent manualized treatment developed by Ehrenreich-May et al. [13] to address different emotional disorders in adolescents. Its goal is to improve emotion reactivity and emotion regulation and to reduce anxiety and depressive symptoms by means of evidence-based treatment techniques (e.g., psychoeducation) that are applied to different emotions including fear, anxiety, sadness, and anger [33]. Studies have shown the efficacy of UP-A in treating anxiety and depressive disorders and symptoms in adolescents [12, 40].

An emerging model of service delivery via Internet has the potential to improve access to evidence-based treatments for anxiety and depressive disorder [5, 39]. This is supported by media compensation theory. This theory provides a new Darwinian framework for understanding and studying electronic communication and teamwork in organizations [19]. Patients in internet-based interventions may receive therapist support faster than in traditional face-to-face treatments. Several meta-analyses have shown that internet-based cognitive-behavioral protocols can be effective in reduction of anxious and depressive symptoms [16]. An internet-based version of UP-A with high feasibility and acceptability was recently developed by Sandín et al. [33] that provides a new approach to improved access to treatment for anxious and depressive adolescents. There is no other study that has delivered the UP-A online for treatment of anxious and depressed adolescents. Furthermore, we found no study on the effectiveness of UP-A (online or face-to-face) in improving psychological flexibility of anxious and depressed adolescents. Before implementation of the intervention, due to the intensification of the Covid-19 pandemic and increasing number of infected individuals) Approximately 95 thousand infected and 6 thousand dead), two lockdown periods had been imposed and schools resumed their educational programs online. During the implementation of the intervention, which coincided with the second peak of the Corona virus epidemic in the world, the number of infected people increased in Iran, and level 3 lockdown was implemented in the country. This was one of the reasons for the necessity of online treatment for adolescents with emotional disorders who were considered vulnerable to this epidemic. After the completion of the treatment, the number of patients decreased for a period of time; but this number increased again, leading to the third COVID peak. The three-month follow-up phase was also implemented during the fourth peak period of this pandemic in the country. In this regard this study aims to investigate the effect of internet-based UP-A intervention on anxiety, depression, stress, and psychological flexibility of adolescents with subclinical features of emotional disorder during the COVID-19 pandemic.

### Hypotheses

Based on the aforementioned literature, a number of hypotheses (H_s_) were formulated. More specifically:

H_1_: Internet-based UP-A intervention would be effective in reducing the anxiety symptoms of adolescents with sub-clinical symptoms of emotional disorders.

H_2_: Internet-based UP-A intervention would be effective in reducing the symptoms of depression in adolescents with sub-clinical symptoms of emotional disorders.

H_3_: Internet-based UP-A intervention would be effective in reducing the symptoms of stress in adolescents with sub-clinical symptoms of emotional disorders.

H_3_: TCBT would be effective in improving psychological flexibility of adolescents with pre-clinical symptoms of emotional disorders.

## Methodology

### Study Design and participants

**Fig. 1:**
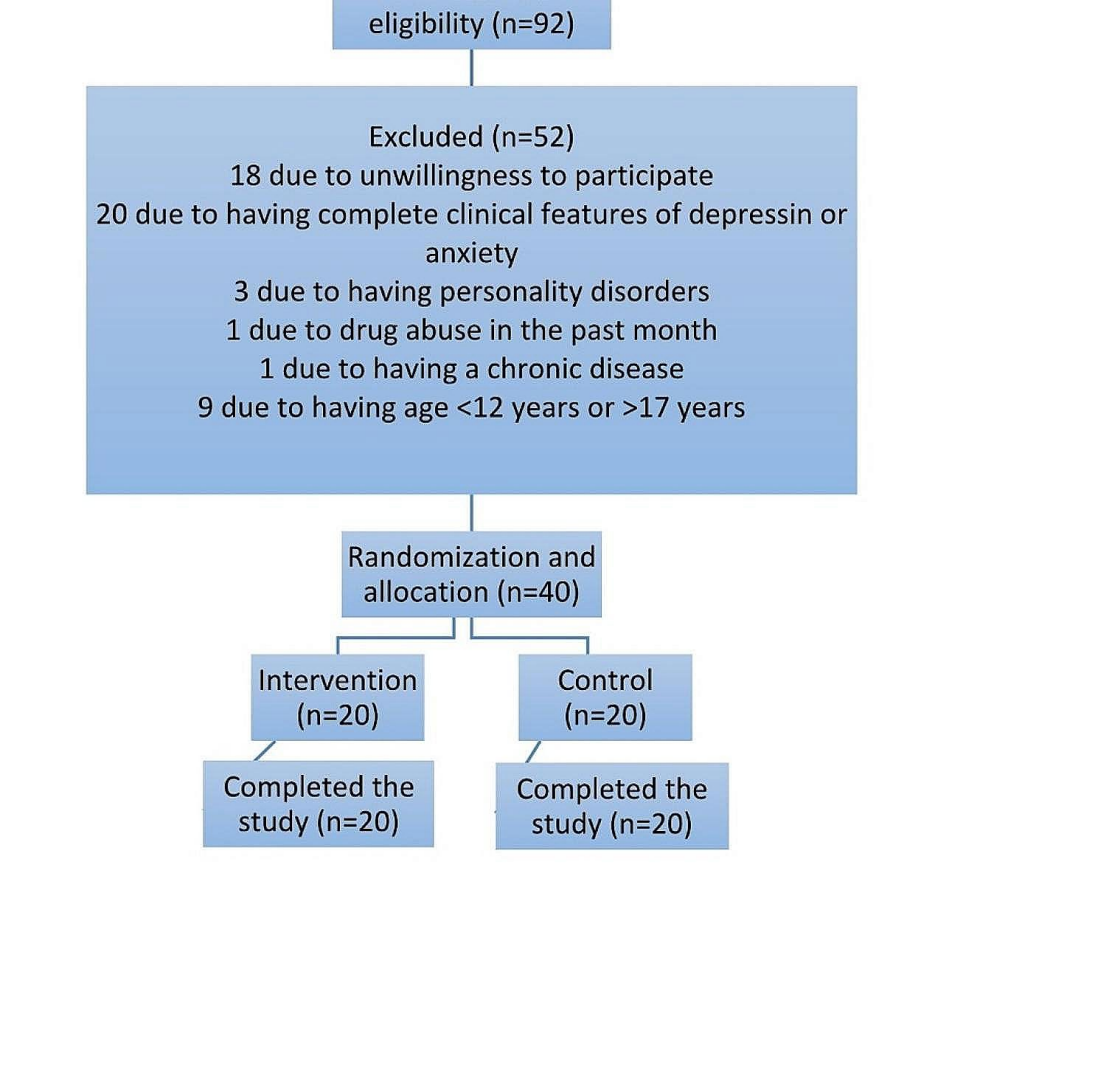
Flowchart of sampling and allocation

This is a longitudinal randomized clinical trial conducted without blinding in 2020 (during the COVID-19 pandemic). The participants were 40 (female and male) adolescents with subclinical features of emotional disorder. They were selected purposively from individuals seeking treatment at Sohravardi and Shafieie as two central specialized clinics of psychiatry and psychology in Zanjan, Iran (*n* = 93) based on inclusion criteria: (i) having subclinical features of emotional disorders according to the results of the Kiddie-Schedule for Affective Disorders and Schizophrenia for School-Age Children-Present and Lifetime (K-SADS-PL) interview and the 21-item Depression Anxiety Stress Scale (DASS-21) score (14–20 for depression and 10–14 for anxiety), (ii) being aged between 12 and 17 years, (iii) having no severe psychiatric disorders (e.g., psychosis and bipolar disorder), (iv) having no cognitive-behavioral interventions in the past five years, and (v) having no medication therapy in the past month. The exclusion criteria were: (i) absence for more than two sessions, (ii) receiving another similar psychological intervention or medication therapy simultaneously, (iii) having complete clinical features of emotional disorders, (iv) taking drugs or alcohol in the past three months, (v) having any chronic diseases, (vi) unwillingness to participate, and (vii) recurrence of emotional problems during the intervention that may require another intervention. The sample size was determined as 20 for each group using the following formula:

$$\mathop n\nolimits_{1} =\mathop n\nolimits_{2} =\frac{{4\left( {1 - \mathop \rho \nolimits^{2} } \right){{\left( {{Z_{1 - \alpha /2}}+{Z_{1 - \beta }}} \right)}^2}}}{{\mathop \delta \nolimits^{2} }}$$

using an effect size (δ) of 0.73, α = 0.05, β = 0.20, and ρ = 0.10The excluded samples would have been replaced with eligible ones. The participants were assigned into two groups of intervention (*n* = 20) and control (*n* = 20) randomly using the function RAND () in Excel. Figure 1 plots the flowchart of sampling and allocation.

### Measures

After obtaining a written informed consent from the participants, which was obtained in an online and documented form, the following instruments were used.

The K-SADS-PL is a semi-structured interview designed for children aged 6–18 years [20]; Persian version: Shahrivar et al [35]., and was used to assess emotional disorders. The instrument is based on the on criteria the Diagnostic and Statistical Manual of Mental Disorders fourth edition (DSM-IV) for emotional disorders. The interview comprises questions regarding personal demographic information (such as age, level of education, name and age of siblings), current complaints and how to adapt to family and school, and information about peers. Then for each disorder, several questions with a variable number are asked for each related criteria. Most items of the K-SADS-PL are scored from 0 to 3 (where 0 indicates that there is not enough information, 1 indicates that there are no symptoms, 2 indicates sub-clinical levels of symptoms, and 3 indicates threshold criteria. The remaining items are scored from 0 to 2 (where 0 indicates the lack of information, 1 indicates the absence of symptoms, and 2 indicates the presence of symptoms). A high score in each disorder indicates presence and severity of the disorder.

The DASS-21 was used to assess the emotional states of depression, anxiety and stress (Lovibond & Lovibond., 1995; Persian version: Sahebi et al [31]. It has 21 items, seven for each of the three subscales. Items (e.g., *“I found it difficult to work up the initiative to do things”*) are rated on a four-point Likert scale from 0 (*did not apply to me at all*) to 3 (*applied to me very much*). Score range from 0 to 21 for each subscale and the higher the scores, the greater the depression, anxiety, and stress, respectively. In the present study, only the anxiety and depression subscales were used. The Cronbach’s alphas were 0.91 for the anxiety subscale and 0.94 for the depression subscale.

Acceptance and Action Questionnaire (AAQ-2) online to have assess psychological flexibility. Developed by Bond et al. [9], the AAQ-2 has 7 items rated on a 7-point Likert scale from 1 (never true) to 7 (always true). Higher total scores mean less flexibility, while lower total scores mean more flexibility. We used the Persian version of AAQ prepared by Abasi et al. [1] who reported its good validity and reliability (0.71–0.89). The two questionnaires were completed before, immediately after, and 3 months after the intervention. It should be mentioned that assessments before and after intervention were performed by an expert in clinical psychology.

### Intervention

The UP-A has 8 treatment modules for adolescents that include building motivation and maintaining it, getting to know emotions and behaviors, introducing emotion-focused behavioral experiments, awareness of body sensations, cognitive flexibility, awareness of emotional experiences, coping with situational emotion exposures, maintaining the gains and one additional module called the “parenting module”. In our study, the intervention group received the UP-A online via video calls on WhatsApp for 2 months at 12 sessions of 45 min (one session for modules 1,3,4,6,8, two sessions for modules 2 and 7, and three sessions for module 5), 2 days per week. It should be noted that the therapist of this study had a master’s degree in clinical psychology who had completed a training course on psychotherapy for adolescents with CBT and transdiagnostic approaches. Moreover, the parents of 8 adolescents who needed this intervention based on the interview results received the parenting module at 1–3 sessions of 45 min for 2–3 weeks, once per week. The control group received no treatment. It should be noted that the control group was waiting list group and did not receive treatment (Table 1).

**Table 1:** Descriptions of the modules in the UP-A and time points

Different courses of intervention and evaluation	Time points Intervention and Control group
**Pre test**	May,2020 Second period of quarantine in Iran
**Intervention (Unified protocol)** **Modules** Module 1: Building motivation and maintaining it Module 2: Getting to know emotions and behaviors Module 3: Introducing emotion-focused behavioral experiments Module 4: Awareness of body sensations Module 5: Cognitive flexibility Module 6: Awareness of emotional experiences Module 7: Coping with situational emotion exposures Module 8: Maintaining the gains Parenting Module	June 2020 Second peak in the world Third period of quarantine in Iran
**Post test**	August 2020 Temporarily reducing the intensity of the corona virus epidemic
**Fallow up (three month after intervention)**	November 2020 fourth peak period in Iran

### Data analysis

The collected data were analyzed in SPSS v.20 using descriptive statistics: frequency (percentage), mean ± standard deviation (SD) and statistical tests including chi-squared test and independent t-test to compare the study groups in terms of gender and age. The Cohen’s d between-group and within-group effect sizes were calculated immediately after and three months after the intervention to make a comparison with a pre-intervention conditions. Furthermore, the marginal longitudinal model was used to compare the groups in terms of DASS-21 subscales and AAQ-2 scores at three evaluation times. The marginal longitudinal model was used because of the departure of anxiety, depression, stress, and psychological flexibility from normality.

## Results

Of the 40 participants, 28 (70.0%) were boys and 12 (30.0%) were girls; 15 boys and 5 girls in the intervention group (Mean ± SD age: 14.2 ± 1.79 years) and 13 boys and 7 girls in the control group (Mean ± SD age: 14.6 ± 1.93 years). The results of chi-squared test for gender (*P* = 0.490) and independent t-test for age (*P* = 0.501) showed that the two groups were homogeneous based on gender and age.

The mean ± SD scores of DASS-21 subscales (depression, anxiety and stress) and AAQ-2 (psychological flexibility) are presented in Table 2. As seen in this table, after the intervention, the anxiety, depression, stress, and psychological flexibility of the patients in the intervention group were reduced (improved). In the control group, anxiety and stress of the participants were increased after the intervention and their depression and psychological flexibility remained almost unchanged. It is likely that the severity of the Covid-19 epidemic was another factor that still prevented the intervention group from achieving a high level of effectiveness. It also made the control group more vulnerable to the severity of anxiety, depression and stress symptoms.

**Table 2:** Mean **±** SD scores of the study variables in the two study groups

Variable	Time	Group		Cohen’s d effect size		
		Intervention Mean ± SD	Control Mean ± SD	Between groups	Within groups*	
**Anxiety**	**Pretest**	14.40 ± 4.33	13.85 ± 4.08	-0.13		
	**Posttest**	11.05 ± 3.50	16.35 ± 4.42	1.33	0.84	0.71
	**Follow-up**	11.70 ± 3.23	16.25 ± 5.24	1.04	-0.59	-0.51
**Depression**	**Pretest**	15.80 ± 5.42	18.30 ± 6.40	0.42		
	**Posttest**	12.85 ± 3.85	18.55 ± 5.33	1.23	0.60	-0.04
	**Follow-up**	12.60 ± 3.76	19.30 ± 5.72	1.38	0.67	0.16
**Stress**	**Pretest**	19.05 ± 4.22	18.55 ± 4.95	-0.11		
	**Posttest**	15.15 ± 3.15	19.60 ± 3.36	1.37	1.02	-0.24
	**Follow-up**	13.30 ± 3.85	18.55 ± 5.35	1.13	1.42	0
**Psychological flexibility**	**Pretest**	24.80 ± 10.68	28.10 ± 10.62	0.31		
	**Posttest**	18.00 ± 7.96	28.40 ± 10.04	0.71	0.69	-0.03
	**Follow-up**	20.35 ± 7.26	29.40 ± 9.89	1.04	0.75	0.13

SD: Standard deviation

*: compared to the pre- intervention stage

After three months, symptoms of emotional disorders of the patients in the intervention group remained almost unchanged, while their stress continued to decrease and their psychological flexibility increased (worsened). In the control group, depression and psychological flexibility of the patients were increased, their anxiety remained almost unchanged, but their stress decreased after three months. Also, the Cohen’s d effect sizes for between-group and within-group comparison are reported in Table 2. Based on these effect sizes, the intervention had improved anxiety, depression, stress, and psychological flexibility in adolescents.

The results of marginal longitudinal model used to compare the two study groups at three time points (before, immediately after, and three months after) for each of the four study variables are reported in Table 3. Also, the estimated mean resulted from fitting marginal model reported in Table 4. As one can see, the estimated mean were the same as the true values. On the other hand, the results of compare within and between groups based on the marginal model in Table 3 are reported in Table 4. There were no significance differences between the two groups before the intervention in terms of anxiety, depression, stress, and psychological flexibility (*P* = 0.672, *P* = 0.172, *P* = 0.725 and *P* = 0.315, respectively). In the stage immediately after the intervention, mean score of the anxiety, depression, stress, and psychological flexibility in the intervention group was significantly lower than the control group (the difference equals to -5.30, -5.70, -4.45, and − 10.40, respectively). Also, three months after the intervention was implemented, the intervention group had lower mean score of the anxiety, depression, stress, and psychological flexibility than the control group. In other words, the mentioned variables have had statistically significant changes, and these changes were maintained during the three months of follow-up (P-value < 0/05. It should also be mentioned that the interaction effect of time and group was significant in relation to these variables, in the sense that over time, the averages have found significant changes in relation to each other).

**Table 3:** Results of marginal longitudinal model for the study variables

Dependent	Independent	Category	regression Coefficient	Standard Error	P-value
**Anxiety**	**Intercept**	**-**	13.85	0.89	< 0.001
	**Time**	**Follow-up**	2.40	1.13	0.033
		**Posttest**	2.50	0.76	0.001
	**Group**	**Intervention**	0.55	1.30	0.672
	Time*Group	Follow-up*intervention	-5.10	1.69	0.003
		Posttest*intervention	-5.85	1.13	< 0.001
**Depression**	**Intercept**	**-**	18.30	1.40	< 0.001
	**Time**	**Follow-up**	1.00	1.25	0.423
		**Posttest**	0.25	0.80	0.755
	**Group**	**Intervention**	-2.50	1.83	0.172
	Time*Group	Follow-up*intervention	-4.20	1.72	0.015
		Posttest*intervention	-3.20	1.21	0.008
**Stress**	**Intercept**	**-**	18.55	1.08	< 0.001
	**Time**	**Follow-up**	-3.72E-16	1.17	> 0.999
		**Posttest**	1.05	0.95	0.270
	**Group**	**Intervention**	0.50	1.42	0.725
	Time*Group	Follow-up*intervention	-5.75	1.54	< 0.001
		Posttest*intervention	-4.95	1.25	< 0.001
**Psychological** **flexibility**	**Intercept**	**-**	28.10	2.31	< 0.001
	**Time**	**Follow-up**	1.30	1.72	0.450
		**Posttest**	0.30	1.33	0.822
	**Group**	**Intervention**	-3.30	3.28	0.315
	Time*Group	Follow-up*intervention	-5.75	2.32	0.013
		Posttest*intervention	-7.10	2.13	0.001

**Table 4:** Estimated mean with comparing results between and within groups based on the results from marginal longitudinal model

Variable	Group	Before	After	Follow-up	P-value (within group)		
					Before - After	Before - Follow-up	After- Follow-up
**Anxiety**	**Control**	13.85	16.35	16.25	0.001	0.033	0.915
	**Intervention**	14.40	11.05	11.70	< 0.001	< 0.001	0.892
	**P-value (between group)**	0.672	< 0.001	0.010			
**Depression**	**Control**	18.30	18.55	19.30	0.755	0.423	0.534
	**Intervention**	15.80	12.85	12.60	0.043	< 0.001	0.039
	**P-value (between group)**	0.172	< 0.001	0.013			
**Stress**	**Control**	18.55	19.60	18.55	0.270	> 0.999	0.314
	**Intervention**	19.05	15.15	13.30	0.010	< 0.001	0.001
	**P-value (between group)**	0.725	< 0.001	0.027			
**Psychological flexibility**	**Control**	28.10	28.40	29.40	0.822	0.449	0.448
	**Intervention**	24.80	18.00	20.35	0.009	< 0.001	0.043
	**P-value (between group)**	0.315	< 0.001	0.028			

## Discussion

The purpose of this clinical trial was to evaluate the effectiveness of internet-based UP-A on stress, anxiety, depression, and psychological flexibility in adolescents with preclinical symptoms of emotional disorders. To our knowledge, this is the first study in Iran and the second study in the world that presented the UP-A online to anxious and depressed adolescents. It was conducted during the COVID-19 pandemic. Based on the results, the intervention could significantly reduce the subclinical symptoms of emotional disorders in adolescents. This is consistent with the results of Sherman et al. [33, 37]. Sherman et al. [37] used the UP-A at 16 sessions on a 15-year-old boy with severe social and generalized anxiety and mild levels of depression. Their results demonstrated significant reductions in anxiety and depressive symptoms, as well as an ability to respond more adaptively to a range of emotional experiences. Sandín et al. [33] used online version of the UP-A in a pilot study for treatment of emotional disorders in 12 adolescents in Spain. Their analyses revealed pre- to post-intervention self-reported decreases of anxiety and depressive symptoms, anxiety sensitivity, emotional avoidance, panic disorder symptoms, panic disorder severity, generalized anxiety disorder symptoms, pathological worry, and major depressive disorder symptoms. Our results are also consistent with the findings of Ehrenreich et al. [11, 12, 36, 40]d Donnell et al. [27]. To explain the result, it can be said that the UP-A probably corrects two major transdiagnostic factors of anxiety sensitivity and emotional avoidance and, thereby, affects the symptoms of emotional disorders. This unified protocol uses various modules (especially modules 3 to 7) to target the common features between anxiety and mood disorders and correct inefficient and pathological strategies used by individuals. By emphasizing how individuals experience and deal with their emotions, it teaches them to respond to their emotions in a more adaptive way. During treatment, efforts are made to improve individuals’ relationship with their emotions by using present-moment and non-judgmental awareness and demonstrating behaviors that are contrary to emotion-focused behaviors, and to modify their behavioral experiments. They practice emotion regulation skills along with situational emotion exposures. These exposures help them tolerate negative feelings.

The internet-based UP-A in our study improved psychological flexibility of anxious and depressed adolescents as well. This is to some extent consistent with the results of Sauer-Zavala et al. [34]. In a controlled trial, they assessed the effect of UP on cognitive flexibility of 88 patients with principal anxiety disorders. After 12–16 treatment sessions, cognitive flexibility of the patients was improved together with their understanding of emotions, mindful-based emotion awareness, countering emotional behaviors, and interoceptive awareness and tolerance. Nasri et al. [25] assessed the effectiveness of UP on cognitive flexibility and emotion regulation of 32 patients with type 2 diabetes, and reported that 12 one-hour sessions of UP could improve their cognitive flexibility and emotion reappraisal. To justify these results, it can be said that under the UP treatment, psychological flexibility of the patients increases once negative automatic thoughts and thinking traps are evaluated and solved. The cognitive flexibility module of this protocol targets these problems. Finally, our result showed that the internet-based UP-A could reduce stress in adolescents with emotional disorders. O’Donnell et al. [27, 41] in their pilot studies showed that 10–14 weeks and 16 weeks of UP treatment significantly reduced posttraumatic stress disorder in patients with posttraumatic psychopathology and veterans, respectively. In a randomized clinical trial, Mohsenabadi et al. [24] reported the efficacy of 12-weekUP treatment in reducing stress of patients with irritable bowel syndrome. These are consistent with our results. It is important to note that in our study, this treatment was implemented during the Covid-19 pandemic and in a situation where all societies, including the country of Iran, were struggling with this source of psychological and social pressure, which definitely affected the participants of the present study. This factor interfered with achieving high quality effectiveness of the treatment and also had an effect on the intensity of the symptoms of emotional disorders in the control group.

There were some limitations in conducting this study. Holding treatment sessions online created some limitations for the therapist and the participants (e.g., it was not possible to send/receive therapy worksheets, there were some distractions). High-speed Internet was sometimes not accessible for the participants, which caused interruptions between treatment sessions. Other limitations of this study include lack of cooperation on the part of some participants (due to having homework or a family member with COVID-19), small sample size, not assessing fidelity, and application of a purposive sampling method. Further studies can be carried out by presenting the UP-A online to assess adolescents using a designed software program, a larger sample size, different follow-up duration, and neuropsychological instruments rather than paper-and-pencil tests.

## Conclusion

Online transdiagnostic therapy based on the UP-A is effective in reducing symptoms of anxiety, depression, stress, and improving psychological flexibility in adolescents during the COVID-19 pandemic. The UP-A can be provided online to improve adolescents’ emotion regulation abilities and psychological flexibility, which can lead to reduced family tensions and improved relationships between parents and children.

## Electronic supplementary material

Below is the link to the electronic supplementary material.

Supplementary Material 1

## Data Availability

The datasets used and analyzed during the present study are available from the corresponding author on reasonable request.
