# Supplementary material for: Internet-based UP-A intervention on treatment of stress, anxiety, depression, and psychological flexibility among adolescents with sub-clinical diagnosis of emotional disorders during the COVID-19 pandemic: a clinical trial
Source: BMC Psychol. 2024 Oct 2;12:526. doi: 10.1186/s40359-024-01735-4 (PMC11448009; doi:10.1186/s40359-024-01735-4)
Supplement: Supplementary file 1 — Supplementary Material 1 [file 40359_2024_1735_MOESM1_ESM.docx]

**Descriptions of the modules in the Unified Protocol for Adolescents (UP-A)**

| Module title | Main contents |
| --- | --- |
| Module 1: Building motivation and maintaining it | This modal emphasizes increasing the readiness and motivation of clients to change their behavior, strengthening self-efficacy and belief in their ability to change successfully, and provides them with an opportunity to weigh the advantages and disadvantages of the change against the behavior they were doing before. Clients are also asked to evaluate treatment goals, plan more objective goals, and identify possible steps to achieve these goals. This part of the treatment is based on the principles and techniques used in motivational interviewing. |
| Module 2: Getting to know emotions and behaviors | The main content of this section is psychoeducational about the nature of emotions, the main components of emotional experience and the concept of learned responses. During this part, it is expected that the client will become more aware of response patterns including perpetuating factors (such as common triggers or environmental dependencies) by searching their emotional experiences. |
| Module 3: Introducing emotion-focused behavioral experiments | This modal emphasizes the behavioral components of emotional experience. In this modal, the therapist helps clients to identify emotional avoidance practices and maladaptive behaviors caused by emotion. After the clients gain more knowledge of how these behaviors affect the continuation of distress, they work with the therapist on changing the current patterns of emotional responses. |
| Module 4: Awareness of body sensations | This modal is designed to help clients identify how to react and respond to their emotions, practice non-judgmental and present-based awareness in emotional experiences. In this modal, patients are expected to acquire the skills of observing emotional experiences. These skills allow them to better understand the thoughts, body sensations and behaviors that contribute to their discomfort. These skills are possible by practicing mindfulness and emotional induction. |
| Module 5: Cognitive flexibility | In this modal clients are taught to pay attention to the role of inconsistent self-evaluations in the emergence of emotional experiences. In this modal, clients learn to identify their thinking patterns, learn ways to adjust maladaptive thinking patterns, and increase their flexibility in evaluating different situations. |
| Module 6: Awareness of emotional experiences | In this modal therapist performs a number of anxiety coping exercises to elicit bodily sensations similar to those associated with anxiety and distress. The purpose of performing these exercises is to identify the role of body sensations in thoughts and behaviors and vice versa. During the confrontation with the physical sensations of anxiety, clients increase their tolerance towards these sensations. |
| Module 7: Coping with situational emotion exposures | In these exposure sessions, the emphasis is on the emotional experience that is created in the real situation. The therapist gives the necessary training in relation to the exposure for situational emotions. Then, during the session, therapist do exposure with the client, and other exposure for home learning are also determined. |
| Module 8: Maintaining the gains | This modal includes an overview of treatment content and clients treatment. The therapist helps the patient identify ways to sustain therapeutic benefits and anticipate future problems. In this modal, the client is encouraged to use therapeutic techniques to achieve short-term and long-term goals. |
| Parenting Module | The purpose of this module is to teach parents how to react to adolescent distress, to teach four common emotional parenting behaviors and their opposite parenting behaviors. |
